# Early Prophylactic Hydrocortisone and Bronchopulmonary Dysplasia–Free Survival in Extremely Preterm Infants

**DOI:** 10.1001/jamanetworkopen.2025.60146

**Published:** 2026-02-19

**Authors:** Veronica Smedbäck, Lars J. Björklund, Anders Flisberg, Jolanta Wróblewska, Olivier Baud, Erik Wejryd, Ulrika Ådén

**Affiliations:** 1 Department of Biomedical and Clinical Sciences, Division of Children’s and Women’s health, Linköping University, Linköping, Sweden; 2 Futurum, Ryhov County Hospital, Jönköping, Sweden; 3 Pediatrics, Department of Clinical Sciences, Lund, Lund University, Lund, Sweden; 4 Department of Pediatric Surgery and Neonatology, Skåne University Hospital, Lund and Malmö, Sweden; 5 Institution of Clinical Sciences, Department of Pediatrics, Sahlgrenska Academy, Gothenburg, Sweden; 6 The Queen Silvia Children’s Hospital, Department of Neonatology, Region Västra Götaland, Sahlgrenska University, Gothenburg, Sweden; 7 Department of Pediatrics, Umeå University, Umeå, Sweden; 8 Obstetric, Perinatal, Paediatric and Life Course Epidemiology Team (OPPaLE), Center for Research in Epidemiology and StatisticS (CRESS), Institut national de la santé et de la recherche médicale (INSERM), Paris Cité University, Paris, France; 9 Department of Neonatal Medicine and Neonatal Intensive Care Unit, FHU Prem’IMPACT, Cochin Port-Royal University Hospital, Assistance Publique-Hôpitaux de Paris, Paris Cité University, 75014, Paris, France; 10 Paris Cité University, Neuro-Diderot, INSERM, Paris, France; 11 Department of Pediatrics, Vrinnevi hospital, Norrköping, Sweden; 12 Department of Women´s and Children´s Health, Karolinska Institutet, Stockholm, Sweden; 13 Department of Neonatal Medicine, Astrid Lindgrens Children´s Hospital. Karolinska University Hospital, Stockholm, Sweden; 14 Department of Neonatal Medicine, H.K.H Crown Princess Victoria Children´s Hospital, Linköping University Hospital, Linköping, Sweden

## Abstract

**Importance:**

In randomized trials, early prophylactic hydrocortisone improved survival without bronchopulmonary dysplasia (BPD) with few adverse effects in extremely preterm infants. Large scale implementation data are needed to evaluate clinical effects and safety.

**Objective:**

To examine the association between early prophylactic hydrocortisone and survival without BPD at 36 weeks’ postmenstrual age (PMA) in extremely preterm infants in Sweden after guideline implementation and to assess treatment safety.

**Design, Setting, and Participants:**

A national historical cohort study with prospectively collected data from the Swedish Neonatal Quality register from 4 Swedish centers where hydrocortisone prophylaxis was implemented. The study included infants born between 22 and 27 weeks’ gestation between 2018 and 2023. Infants were divided into exposed and nonexposed groups according to the intention-to-treat principle.

**Exposure:**

Hydrocortisone, 1 mg/kg/d, for the first 7 days of life, followed by 0.5 mg/kg/d from days 8 through 10.

**Main outcomes and measures:**

The primary outcome was survival without BPD at 36 weeks’ PMA. A predefined statistical analysis plan with logistic regression was used to calculate unadjusted and adjusted odds ratios.

**Results:**

Among 1106 infants (median [IQR] gestational age, 25 weeks, 6 days [24 weeks, 3 days to 27 weeks]; median [IQR] birth weight, 780 [610-964] g), 474 received hydrocortisone prophylaxis and 632 did not. Survival without BPD occurred in 154 of 474 exposed (32.5%) and 185 of 632 nonexposed (29.3%) infants (adjusted odds ratio, 1.62; 95% CI, 1.16-2.27). BPD occurred in 233 exposed (49.2%) and 345 nonexposed (54.6%) infants (adjusted odds ratio, 0.65; 95% CI, 0.49-0.86). Death before 36 weeks’ PMA occurred in 87 exposed (18.4%) and 102 nonexposed (16.1%) infants. Late-onset bacterial infection was more common in exposed infants, but not significant after adjustment. No other severe neonatal morbidities differed significantly between the 2 groups.

**Conclusions and relevance:**

In this cohort study of extremely preterm infants, the introduction of prophylactic hydrocortisone was associated with increased survival without BPD, after adjusting for covariates. There was no significant increase in severe neonatal morbidities, except that late-onset bacterial infection was more common in the exposed group before adjustments.

## Key Points

**Question:**

After guideline implementation, has early prophylactic hydrocortisone improved survival without bronchopulmonary dysplasia (BPD) in extremely preterm infants born in Sweden, and is it safe to use?

**Findings:**

In this cohort study using prospectively collected data from 1106 infants from a national register, introduction of prophylactic hydrocortisone was followed by an increased likelihood of survival without BPD. There was no significant increase in severe neonatal morbidities.

**Meaning:**

These findings, based on clinical implementation data, suggest alignment with previous similar studies supporting the benefits and safety of early prophylactic hydrocortisone treatment.

## Teaser

This cohort study evaluates the association between early prophylactic hydrocortisone and survival without bronchopulmonary dysplasia (BPD) in extremely preterm infants in Sweden.

## Introduction

Bronchopulmonary dysplasia (BPD) is a common chronic lung disease among infants born extremely preterm, defined as birth before 28 weeks’ gestation. Reported incidence rates vary widely, and in Sweden, approximately 50% to 60% of infants born extremely preterm are diagnosed with BPD at 36 weeks’ postmenstrual age (PMA).^1,2,3^ The multifactorial pathogenesis of BPD involves inflammation and is often associated with mechanical respiratory support. Extremely preterm infants are particularly vulnerable due to immature adrenal function, low endogenous cortisol and exposure to postnatal stressors commonly encountered in the neonatal intensive care unit increasing the risk of developing BPD.^4,5,6^

Numerous studies have indicated that corticosteroid replacement therapy may offer benefits for extremely preterm infants by supporting hemodynamic stability, promoting lung development and reducing inflammation.^7^ Dexamethasone has historically been used to prevent or treat BPD, but early intervention is associated with increased risk of short- and long-term adverse effects, including cerebral palsy.^8,9^

Previous trials have documented positive effects of early prophylactic hydrocortisone on lung development and mortality without causing neurodevelopmental impairment.^10,11,12,13^ The effect of prophylactic hydrocortisone in extremely preterm infants in clinical practice has only previously been reported in 3 smaller single center studies.^11,14,15^ Thus, population-based clinical data are needed to further assess its effectiveness. Moreover, the treatment has been associated with adverse events, such as bowel perforation and late-onset sepsis, complications that may be related to other treatment interactions or clinical practices such as concurrent use of indomethacin.^16,17,18^ Consequently, the safety aspects of prophylactic hydrocortisone need to be further studied and evaluated in clinically representative settings.

This study aimed to analyze the association of early prophylactic hydrocortisone use in Sweden with changes in the incidence of survival without BPD in extremely preterm infants. Our hypotheses were that the introduction of prophylactic hydrocortisone would be followed by an increased survival without BPD at 36 weeks’ PMA but not by an increased rate of adverse events or severe neonatal morbidities.

## Methods

### Design and Cohort

This study was a historical cohort study using prospectively collected data. Eligible infants were born before 28 weeks’ gestation and admitted to the neonatal intensive care unit on the first day of life at a hospital where prophylactic hydrocortisone had been implemented (South, West, North, and Southeast centers of Sweden). No exclusion criteria were applied. Data from January 2018 to December 2023 were collected from the Swedish Neonatal Quality Register (SNQ), a national database prospectively recording daily clinical information covering 98% of infants admitted to neonatal care in Sweden.^19^ Two sensitivity analyses were made: one using a broader national cohort including all extremely preterm infants in Sweden during 2018 to 2023, regardless of center, and one using 1:1 propensity score–matched groups which was applied in both cohorts. Infants were divided into exposed and nonexposed groups according to the intention-to-treat principle across all analyses. Infants born before the implementation date were assumed to be nonexposed, whereas infants born on or after the implementation date were assumed to be exposed to the hydrocortisone prophylaxis if born at a center where the guideline was implemented. Thus, the main analysis used historical controls, whereas sensitivity analyses in the larger cohort included both historical and contemporary controls. The study followed the Strengthening the Reporting of Observational Studies in Epidemiology (STROBE) reporting guideline for observational studies.^20^ The Swedish Ethical Review Authority approved the study and granted a waiver of informed consent.

### Exposure

The exposure investigated in this study was prophylactic hydrocortisone administered intravenously as hydrocortisone sodium succinate, 0.5 mg/kg, twice daily from days 1 to 7 of life, followed by 0.5 mg/kg once daily for days 8 to 10, yielding a cumulative dose of 8.5 mg/kg. Oral administration was used when intravenous access was unavailable, typically toward the end of treatment. In 3 of the 4 centers, the treatment was given to all admitted infants born before 28 weeks’ gestation. In 1 center (the North), the treatment was given to all infants born before 26 weeks’ gestation and to infants born at 26 weeks, 0 days’ to 27 weeks, 6 days’ gestation if the mother had suspected chorioamnionitis. The validated dates of implementation by local physicians were September 1, 2020, in the Western center; January 1, 2021, in the Southern and Northern centers; and April 1, 2022, in the Southeastern center. The 2 remaining centers in Sweden (East and Middle) chose not to adopt this treatment protocol.

### Outcomes

The primary outcome was survival without BPD at 36 weeks’ PMA. BPD was defined as the need for supplemental oxygen at 36 weeks’ PMA. Subgroup analyses were made from the components of primary outcome in terms of BPD incidence and mortality before 36 weeks’ PMA. Stratifications of primary outcome by gestational age (GA), birth weight, chorioamnionitis, and sex were also made as subgroup analyses. Chorioamnionitis was defined by maternal fever with clinical or microbiological signs of infection or histopathologic placental inflammation. Secondary outcomes included age at the last day on mechanical ventilation, age at the last day on respiratory support including mechanical ventilation, continuous positive airway pressure, high flow nasal cannula, and any extra oxygen. Other secondary outcomes were pharmacologic or surgical treatment for patent ductus arteriosus, and the use of other systemic steroids. Safety outcomes included short term severe neonatal morbidities as defined by the register, including spontaneous intestinal perforation, necrotizing enterocolitis, late bacterial infection with positive cultures, pulmonary hemorrhage, insulin treatment, retinopathy of prematurity (ROP) diagnosis, treatment for ROP, intraventricular hemorrhage grade 3 to 4, and cystic periventricular leukomalacia.

### Statistical Analysis

All analyses followed a prespecified statistical analysis plan created by the authors and statisticians. For descriptive analyses, Fisher exact tests were used for dichotomous variables, χ^2^ tests for categorical variables, and *t* tests or Mann-Whitney U-tests for continuous variables, depending on the distribution of the variable. Logistic regression was performed as the main analysis, both unadjusted and adjusted for relevant covariates, with odds ratios (OR) calculated along with 95% CIs and *P* values. Covariates with significant association with the primary outcome were included in the adjusted models.

Sensitivity analyses were conducted using 1:1 propensity score matching based on all baseline characteristics, allowing balanced group comparisons without adjustments for covariates. A nearest neighbor matching algorithm with a caliper width of 0.2 of the SD of the logit was applied with validation based on distribution scores between exposed and nonexposed groups before matching.

Continuous outcome variables were analyzed using linear regression, assuming normal distribution when applicable or negative binomial regression depending on the distribution of the variables. Results from the negative binomial regression are presented as risk ratios with 95% CIs and *P* values. An analogous analysis to interrupted time series was performed using logistic regression to investigate the effect of a time variable on the primary outcome with estimated changes in odds per 1-year increase in birth year between the groups. The number needed to treat to achieve survival without BPD was calculated. All tests were 2-tailed and conducted at a significance level of .05. Analyses were completed in SAS version 9.4 (SAS Institute).

## Results

### Cohort Characteristics

According to SNQ, 2011 infants were born extremely preterm in Sweden between January 2018 and December 2023. Of these, 1140 were born in centers where prophylactic hydrocortisone was implemented. A total of 1106 infants were included while 34 were excluded due to later admission than first day of life or missing data in the register. Infants were divided into groups of exposed and nonexposed to prophylactic hydrocortisone according to the intention-to-treat principle as described in the Methods section (Figure). The sensitivity analyses applied the same principle, including analyses using 1:1 propensity score–matched groups. The GA was significantly lower in the exposed group (median [IQR] GA, 25 weeks, 4 days [24 weeks, 2 days to 27 weeks, 0 days]) compared with the control group (median [IQR] 26 weeks, 1 day [24 weeks, 4 days to 27 weeks, 1 day]; mean difference, −0.21; 95% CI, −0.40 to 0.01; *P* = .02) (Table 1). No other baseline characteristics differed significantly. The median (IQR) GA for the whole cohort was 25 weeks, 6 days (24 weeks, 3 days to 27 weeks, 0 days) and median (IQR) birth weight was 780 (610-964) grams. All baseline characteristics are shown in Table 1. Baseline characteristics from the sensitivity analyses are detailed in eTable 1 in Supplement 1 for the propensity score–matched groups and in eTable 2 in Supplement 1 for the larger national cohort.

**Figure.**
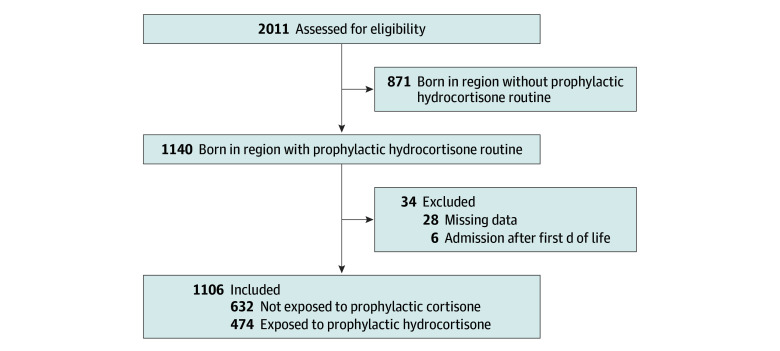
Flowchart of the Cohort

**Table 1.** Baseline Characteristics and Group Comparisons of Exposed and Nonexposed Infants

Baseline characteristics	Participants, No. (%)		*P* value^a^
	Exposed group (N = 474)	Nonexposed group (N = 632)	
GA, wk			
Median (IQR)	25 wk, 4 d (24 wk, 2 d to 27 wk, 0 d)	26 wk, 1 d (24 wk, 4 d to 27 wk, 1 d)	.02
22-23	92 (19.4)	97 (15.3)	
24-25	180 (38.0)	213 (33.7)	
26-27	202 (42.6)	322 (50.9)	
Birth weight (IQR), g	755 (603-950)	800 (610-968)	.23
Sex			
Female	217 (45.8)	286 (45.3)	.90
Male	257 (54.2)	346 (54.7)	
Prenatal steroids given, No./total No. (%)	425/452 (94.0)	536/589 (91.0)	.08
Surfactant given	365 (77.0)	470 (74.4)	.32
Multiple birth	99 (20.9)	159 (25.2)	.18
Intubation at birth, No./total No. (%)	185/463 (40.0)	228/621 (36.7)	.28

Abbreviation: GA, gestational age.

^a^ For test between 2 groups, Fisher exact test was used for dichotomous variables, χ^2^ test for categorical variables, and Mann-Whitney U-test for continuous variables based on the variable distribution.

### Main Findings

#### Primary Outcome

In the exposed group, 154 of 474 infants (32.5%) survived without BPD, compared with 185 of 632 infants (29.3%) in the nonexposed group. The unadjusted OR was 1.16 (95% CI, 0.90-1.50), and adjusted OR (aOR) was 1.62 (95% CI, 1.16-2.27) (Table 2). Covariates that were significantly associated with the primary outcome were GA (weeks) and birth weight in grams, as well as z-score, sex, multiple births, prenatal steroids, Apgar at 10 minutes, intubation at birth, center of birth, and surfactant treatment (see eTable 3 in Supplement 1). These were included in the adjusted logistic regression except when the covariate also was a subgroup in the analysis.

**Table 2.** The Primary Outcome and Its Components, With and Without Stratification

Outcome	Exposed group (n = 474)	Control group (n = 632)	OR (95% CI)^a^	*P* value^a^	aOR (95% CI)^a^	*P* value^a^
Survival without BPD	154 (32.5)	185 (29.3)	1.16 (0.90-1.50)	.25	1.62 (1.16-2.27)	.01
Components of the primary outcome						
BPD at 36 wk PMA	233 (49.2)	345 (54.6)	0.80 (0.63-1.02)	.07	0.65 (0.49-0.86)	.002
Death before 36 wk PMA	87 (18.4)	102 (16.1)	1.17 (0.85-1.60)	.33	1.12 (0.75-1.69)	.58
Survival without BPD stratified by GA^b^						
22-23 wk	5 (5.4)	4 (4.1)	1.34 (0.35-5.14)	.67	NA^c^	NA^c^
24-25 wk	44 (24.4)	29 (13.6)	2.06 (1.23-3.47)	.01	NA^c^	NA^c^
26-27 wk	105 (52.0)	152 (47.2)	1.20 (0.85-1.71)	.30	1.30 (0.85-1.99)	.23
Survival without BPD stratified by birthweight deviation^b^						
Small for GA	24 (24.2)	30 (22.9)	1.08 (0.58-1.99)	.81	1.44 (0.73-2.82)^d^	.29^d^
Appropriate birth weight	129 (34.8)	152 (31.0)	1.19 (0.89-1.58)	.24	1.62 (1.1-2.35)	.01
Survival without BPD stratified by chorioamnionitis^b^						
Chorioamnionitis	17 (34.0)	9 (20.0)	2.06 (0.81-5.25)	.13	7.78 (1.72-35.14)^d^	.01^d^
No chorioamnionitis	137 (32.3)	176 (30.0)	1.11 (0.85-1.46)	.43	1.52 (1.08-2.15)	.02
Survival without BPD stratified by sex^b^						
Female	79 (36.4)	95 (33.2)	1.15 (0.79-1.67)	.46	1.57 (0.96-2.57)	.07
Male	75 (29.2)	90 (26.0)	1.17 (0.82-1.68)	.39	1.65 (1.03-2.64)	.04

Abbreviations: aOR, adjusted odds ratio; BPD, bronchopulmonary dysplasia; GA, gestational age; OR, odds ratio; PMA, postmenstrual age.

^a^ Logistic regression was used unadjusted and adjusted for covariates. The following covariates were used to adjust, except when covariate is a subgroup: sex, multiple births, birth weight with and without z-score, GA (weeks), Apgar score at 10 minutes, intubation at birth, center of birth, prenatal steroids, and surfactant.

^b^ Not adjusted for center of birth.

^c^ Not enough events to make adjusted logistic regression analysis possible.

^d^ Only adjusted for GA.

Sensitivity analyses were repeated with 1:1 propensity score–matched groups with an OR of 1.40 (95% CI, 1.04-1.89) for survival without BPD between exposed and nonexposed groups (eTable 4 in Supplement 1). In the larger national cohort including all 6 Swedish centers, the OR for survival without BPD was 1.01 (95% CI, 0.81-1.26) and the aOR was 1.58 (95% CI, 1.14-2.20) (eTable 5 in Supplement 1).

The number needed to treat to achieve 1 additional infant surviving without BPD was 32 based on unadjusted incidence rates and 15 using the propensity score–matched groups. Analyzing the components in the primary outcome (BPD and mortality before 36 weeks’ PMA) yielded an OR of 0.80 (95% CI, 0.63-1.02) for BPD between the 2 groups and an aOR of 0.65 (95% CI 0.49-0.86) (Table 2). There was no significant difference between the exposed and nonexposed groups in mortality before 36 weeks’ PMA. Death before 36 weeks’ PMA occurred in 87 exposed (18.4%) and 102 nonexposed (16.1%) infants.

Stratified analysis by GA of primary outcome showed that the outcome was most pronounced in infants born at 24 to 25 weeks’ gestation (OR, 2.06; 95% CI, 1.23-3.47) (Table 2). Due to few eligible and included infants, adjusted regression could not be performed in this subgroup, but propensity score-matched analysis yielded an OR of 2.51 (95% CI, 1.33-4.75). Additional subgroup analyses by birth weight, presence of chorioamnionitis, and sex are shown in Table 2. In the national cohort, similar reductions in OR for BPD were observed (aOR, 0.66; 95% CI, 0.50-0.87), as well as increased survival without BPD in infants born at 24 to 25 weeks’ gestation (OR, 1.56; 95% CI, 1.03-2.35) (eTable 5 in Supplement 1). Analogous interrupted time series analysis did not show any significant difference in ORs or aORs between the exposed and nonexposed group and is presented in eTable 6 in Supplement 1.

#### Secondary Outcomes

There were no significant differences between the exposed and nonexposed groups in the incidence of PDA, neither overall nor in subgroups defined by need of medical or surgical treatment for PDA. This finding remained consistent across unadjusted and adjusted logistic regression, as well as in propensity score–matched analyses (Table 3).

**Table 3.** Secondary Outcomes

Respiratory outcomes^a^	Exposed group (n = 474)	Control group (n = 632)	RR (95% CI)	*P* value	aRR (95% CI)	*P* value
Age at last day on respiratory support, mean (95% CI), d^b^	77 (73-83)	73 (69-77)	1.07 (0.98-1.16)	.13	1.03 (0.95-1.11)	.54
Age at last day on mechanical ventilation, mean (95% CI), d	33 (29-38)	34 (30-38)	0.98 (0.82-1.17)	.85	0.97 (0.81-1.16)	.73
Days on mechanical ventilation, mean (95% CI)	12 (10-14)	12 (11-14)	0.96 (0.79-1.18)	.73	0.77 (0.63-0.93)	.01
Other secondary outcomes^c^			OR (95% CI)		aOR (95% CI)	
Patent ductus arteriosus, medically treated, No. (%)	218 (46.0)	278 (44.0)	1.08 (0.85-1.38)	.51	0.89 (0.67-1.19)	.45
Patent ductus arteriosus, surgically treated, No. (%)	26 (5.5)	39 (6.2)	0.88 (0.53-1.47)	.63	0.76^d^ (0.45-1.30)	.32^d^
Administration of systemic steroids other than prophylactic hydrocortisone, No. (%)	128 (27.0)	191 (30.2)	0.85 (0.66-1.11)	.24	0.77 (0.56-1.05)	.10

Abbreviations: aRR, adjusted relative risk; RR, relative risk.

^a^ Negative binomial regression was used unadjusted and adjusted for covariates.

^b^ Respiratory support includes mechanical ventilation, continous positive air pressure, high flow nasal cannula, and any other extra oxygen.

^c^ Logistic regression was used unadjusted and adjusted for covariates. The following covariates were used to adjust for negative binomial and logistic regression: sex, multiple births, birth weight with and without z-score, gestational age (weeks), Apgar score at 10 minutes, intubation at birth, center of birth, prenatal steroids, and surfactant.

^d^ Only adjusted for gestational age.

Infants in the exposed group had significantly fewer days on mechanical ventilation compared with nonexposed infants after adjustments (adjusted relative risk, 0.77; 95% CI, 0.63-0.93) (Table 3). No significant differences were observed in the unadjusted analysis or in other respiratory variables, including age at last day on respiratory support. The use of systemic corticosteroids other than prophylactic hydrocortisone did not differ significantly between the 2 groups (aOR, 0.77; 95% CI, 0.56-1.05) (Table 3).

### Safety Analyses

There were no significant differences between exposed and nonexposed groups in the incidence of spontaneous intestinal perforation, necrotizing enterocolitis, insulin treatment, pulmonary hemorrhage, intraventricular hemorrhage, treatment for ROP, or cystic periventricular leukomalacia (Table 4). Late-onset bacterial infection occurred more frequently in the group exposed to prophylactic hydrocortisone compared with the nonexposed group (23.8% vs 17.7%; OR, 1.45; 95% CI, 1.08-1.95), but this difference was no longer statistically significant after adjustment for covariates (aOR, 1.30; 95% CI, 0.93-1.82) (Table 4). No significant differences were observed in propensity score–matched analyses, although late-onset bacterial infection was close to statistically significant (OR, 1.39; 95% CI, 0.99-1.94) (eTable 7 in Supplement 1). In the larger national cohort, both late-onset bacterial infection and insulin treatment were more common in the exposed group; however, no safety outcomes were significant after adjustments or propensity score matching (eTable 8 in Supplement 1).

**Table 4.** Outcome of Safety Variables

Safety variables	Exposed group (n = 474)	Control group (n = 632)	OR (95% CI)^a^	*P* value^a^	aOR (95% CI)^a^	*P* value^a^
Pulmonary hemorrhage	10 (2.1)	18 (2.8)	0.74 (0.34-1.61)	.44	0.73 (0.33-1.60)^b^	.43^b^
Spontaneous intestinal perforation	3 (0.6)	7 (1.1)	0.57 (0.15-2.21)	.42	NA^c^	NA^c^
Insulin treatment	111 (23.4)	134 (21.2)	1.14 (0.85-1.51)	.38	1.05 (0.73-1.50)	.79
Late-onset sepsis	113 (23.8)	112 (17.7)	1.45 (1.08-1.95)	.01	1.30 (0.93-1.82)	.13
Necrotizing enterocolitis	50 (10.5)	53 (8.4)	1.29 (0.86-1.93)	.22	1.28 (0.81-2.01)	.29
IVH grade 3 or 4	61 (14.0)	83 (14.0)	1.00 (0.70-1.43)	.99	0.95 (0.63-1.44)	.82
ROP, any grade	178 (37.6)	234 (37.0)	1.02 (0.80-1.31)	.86	1.00 (0.76-1.33)	.98
Treatment for ROP, grade 3-5	41 (8.6)	54 (8.5)	1.01 (0.66-1.55)	.95	1.05 (0.64-1.70)	.86

Abbreviations: aOR, adjusted odds ratio; IVH, intraventricular hemorrhage; NA, not applicable; OR, odds ratio; ROP, retinopathy of prematurity.

^a^ Logistic regression was used unadjusted and adjusted for covariates. The following covariates were used to adjust: sex, multiple births, birth weight with and without z-score, gestational age (weeks), Apgar score at 10 minutes, intubation at birth, center of birth, prenatal steroids, and surfactant.

^b^ Only adjusted for gestational age due to few events.

^c^ Not enough events for analysis.

## Discussion

In this national historical cohort study, based on prospectively collected register data, the implementation of early prophylactic hydrocortisone in extremely preterm infants was associated with significantly increased survival without BPD after adjustments. This benefit was not accompanied by an increased risk of severe neonatal morbidities.

Late-onset bacterial infection was more common in the exposed group in unadjusted analyses, consistent with findings from the Trial to Prevent Bronchopulmonary Dysplasia in Very Preterm Neonates (PREMILOC) and meta-analysis.^10,16^ After adjustments and using propensity score–matched analyses, the difference was no longer significant, although a trend persisted. Shah et al^11^ also investigated the safety of prophylactic hydrocortisone in extremely preterm infants and similarly found no significant association with rates of positive bacterial cultures and prophylactic hydrocortisone.

Neither our study nor that of Shah et al^11^ identified an association between prophylactic hydrocortisone and bowel perforation, despite prior reports suggesting such a link in certain trials.^13,17,18,21^ Limited use of indomethacin in Sweden may explain this finding since indomethacin treatment for PDA has been implicated in increasing the risk of bowel perforation.^16,22^

To minimize confounding, we used both historical controls from centers that implemented hydrocortisone prophylaxis and a national cohort with contemporary controls added from centers that have not implemented the treatment. This reduced bias from differences in clinical practice as well as the potential impact of improvements in clinical practices over time. In both strategies, propensity score matching was applied as a sensitivity analysis, and findings remained consistent after adjustments. This persistence of similar outcomes strengthens our findings and conclusions. Our additional analysis from eTable 6 in Supplement 1, including a time variable, showed no significant temporal trend or difference between the groups, supporting the interpretation that the implementation of hydrocortisone prophylaxis was the key factor associated with the observed increase in survival without BPD.

Previous randomized trials have shown reduced mortality with early prophylactic hydrocortisone, with mixed effects on BPD.^8^ However, both our findings and those of Shah et al suggest that the main driver of increased survival without BPD is a reduction in BPD incidence, rather than improved survival. One speculation is that this difference may reflect overall improvements in neonatal care and lower baseline mortality in recent years, making it more difficult to demonstrate a mortality benefit.^2,23,24^

The timing of prophylactic hydrocortisone may also play a crucial role. Previous studies have shown that the fetal adrenal gland continues to mature until full term, and that extreme preterm birth is associated with cortisol insufficiency. Therefore, the effectiveness of hydrocortisone replacement may depend on the timing of exposure.^25,26^ We observed a stronger association between prophylactic hydrocortisone and increased survival without BPD in infants born at 24 to 25 weeks of gestation. This more pronounced improvement in these infants supports the hypothesis that early low-dose prophylactic hydrocortisone serves as a replacement therapy for the cortisol deficiency caused by immature adrenals.

It is possible that benefits may extend to infants born at 22 to 23 weeks’ gestation; however, our study was underpowered to detect a significant association due to few events resulting in wider confidence intervals. Additionally, this study examined subgroups for the primary outcome with a hypothesis that exposure to prophylactic hydrocortisone would have a greater effect in these subgroups, but this was not always the case and few events may also have limited the statistical power for some of these analyses.

While previous studies have reported that prophylactic hydrocortisone may reduce the need for PDA treatment,^10,11,16^ we did not observe such an association. A possible explanation for this may be the discrepancy in treatment practices for PDA in different countries, which could lead to population-level differences in treatment thresholds or preferences and thereby limit cross-study comparability.^27^

### Strengths and Limitations

This study has several strengths. Use of a high-quality national register with near-complete data capture enabled evaluation in clinical settings using a large multicenter cohort, reducing selection bias and improving generalizability. Inclusion of all participating centers minimized center-related bias. The study further strengthens the evidence base by including a population comparable in size to the PREMILOC trial and prior implementation studies combined, and by providing data on infants born at 22 to 23 weeks’ gestation, a group rarely reported in previous studies.

This study has limitations. The BPD diagnosis was not validated using oxygen reduction tests and was instead defined by the need for supplemental oxygen at 36 weeks’ PMA, which limited classifications including severity. However, this pragmatic definition is widely used in clinical practice. Variation in BPD definitions across prior studies also complicates direct comparisons. Despite these limitations, our findings are broadly consistent with the PREMILOC trial and other randomized and observational studies.^10,11,16^ Assessment of BPD severity may be particularly relevant, given that a recent study reported improved survival without cerebral palsy following dexamethasone exposure in infants with severe BPD.^28^ Thus, the effect of prophylactic hydrocortisone on severe BPD and survival without cerebral palsy remains uncertain.

Use of an intention-to-treat approach may have included infants with incomplete protocol adherence; however, this approach was appropriate for evaluating guideline implementation and reduced selection bias, including otherwise possible exclusions of infants who died before treatment initiation. Additionally, reliance on clinician-reported register data limited independent data verification, although data collection has followed standardized protocols for more than 20 years.^19^

Furthermore, the study emphasizes the need for further research, ideally through prospective studies, to identify whether specific subgroups of extremely preterm infants may derive greater benefit or face harm from early prophylactic hydrocortisone therapy. Additionally, follow-up studies are particularly important to assess the impact on neurodevelopmental outcomes and are currently being planned for this cohort.

## Conclusions

This national cohort study provides evidence that the introduction of early prophylactic hydrocortisone to extremely preterm infants was associated with increased survival without BPD after adjustments, without any registered short-term adverse effects. Long-term follow-up and further prospective studies are warranted to assess potential risks and benefits, particularly regarding neurodevelopmental outcomes and identification of high-risk subgroups.

## Notes

Supplement 1. eTable 1. Baseline Characteristics With Propensity Score–Matched Groups Analyses Presented eTable 2. Baseline Characteristics With Cohort of Infants From all Swedish Regions Born Extremely Preterm, 2018 to 2023 eTable 3. Statistical Evaluation of Confounders to Primary Outcome eTable 4. The Primary Outcome and its Components, With and Without Stratification and With Propensity Score–Matched Groups Analyses Presented eTable 5. The Primary Outcome and Its Components, With and Without Stratification With Cohort on All Infants Born Extremely Preterm, 2018 to 2023 eTable 6. Trend Analysis of Primary Outcome by Date of Birth eTable 7. Outcome on Safety Variables With Propensity Score–Matched Groups Analyses Presented eTable 8. Outcome on Safety Variables With Cohort of Infants From All Swedish Regions Born Extremely Preterm, 2018 to 2023

Supplement 2. Data Sharing Statement
