# Supplementary material for: Early Prophylactic Hydrocortisone and Bronchopulmonary Dysplasia–Free Survival in Extremely Preterm Infants
Source: JAMA Netw Open. 2026 Feb 19;9(2):e2560146. doi: 10.1001/jamanetworkopen.2025.60146 (PMC12921520; doi:10.1001/jamanetworkopen.2025.60146)
Supplement: Supplement 1. — eTable 1. Baseline Characteristics With Propensity Score–Matched Groups Analyses Presented eTable 2. Baseline Characteristics With Cohort of Infants From all Swedish Regions Born Extremely Preterm, 2018 to 2023 eTable 3. Statistical Evaluation of Confounders to Primary Outcome eTable 4. The Primary Outcome and its Components, With and Without Stratification and With Propensity Score–Matched Groups Analyses Presented eTable 5. The Primary Outcome and Its Components, With and Without Stratification With Cohort on All Infants Born Extremely Preterm, 2018 to 2023 eTable 6. Trend Analysis of Primary Outcome by Date of Birth eTable 7. Outcome on Safety Variables With Propensity Score–Matched Groups Analyses Presented eTable 8. Outcome on Safety Variables With Cohort of Infants From All Swedish Regions Born Extremely Preterm, 2018 to 2023 [file jamanetwopen-e2560146-s001.pdf]

Supplemental Online Content

Smedbäck V, Björklund LJ, Flisberg A, et al. Early prophylactic hydrocortisone, bronchopulmonary dysplasia, and survival in extremely preterm infants. *JAMA Netw Open*. 2026;9(2):e2560146. doi:10.1001/jamanetworkopen.2025.60146

eTable 1. Baseline Characteristics With Propensity Score Matched Groups Analyses Presented

eTable 2. Baseline Characteristics With Cohort of Infants From all Swedish Regions Born Extremely Preterm, 2018 to 2023

eTable 3. Statistical Evaluation of Confounders to Primary Outcome

eTable 4. The Primary Outcome and its Components, With and Without Stratification and With Propensity Score–Matched Groups Analyses Presented

eTable 5. The Primary Outcome and Its Components, With and Without Stratification With Cohort on All Infants Born Extremely Preterm, 2018 to 2023

eTable 6. Trend Analysis of Primary Outcome by Date of Birth

eTable 7. Outcome on Safety Variables With Propensity Score Matched Groups Analyses Presented

eTable 8. Outcome on Safety Variables With Cohort of Infants From All Swedish Regions Born Extremely Preterm, 2018 to 2023

This supplemental material has been provided by the authors to give readers additional information about their work.

© 2026 LaMonte MJ et al. *JAMA Network Open*.

eTable 1. Baseline Characteristics With Propensity Score Matched Groups Analyses Presented

Baseline characteristics	Exposed group (N=474)	Non-exposed group (N=632)	p-value	PS 1:1 p-value (N=419/419)
GA, weeks (IQR)	25+4 (24+2 – 27+0)	26+1 (24+4 – 27+1)	0.02	0.49
GA 22-23 weeks, n (%)	92 (19.4%)	97 (15.3%)		
GA 24-25 weeks, n (%)	180 (38.0%)	213 (33.7%)		
GA 26-27 weeks, n (%)	202 (42.6%)	322 (50.9%)		
Birth weight, g (IQR)	755 (603-950)	800 (610-986)	0.23	0.63
Male sex, n (%)	257 (54.2%)	346 (54.7%)	0.90	0.94
Prenatal steroids given, n (%)	425 (94.0%)	536 (91.0%)	0.08	0.32
Surfactant given, n (%)	365 (77.0%)	470 (74.4%)	0.32	0.52
Multiple births, n (%)	99 (20.9%)	159 (25.2%)	0.18	0.56
Intubation at birth, n (%)	185 (40.0%)	228 (36.7%)	0.28	0.89

Data are presented as median (interquartile range) or number (percentage). For test between two groups Fisher's exact test was used for dichotomous variables, Chi-square test for categorical variables, and Mann-Whitney U-test for continuous variables based on the variable distribution.
Following covariates were used to create propensity score matched groups: gestational age (weeks), birth weight, Apgar score 10 min, sex, multiple births, intubation at birth, prenatal steroids, chorioamnionitis and surfactant.
GA Gestational age.

© 2026 LaMonte MJ et al. *JAMA Network Open*.

eTable 2. Baseline Characteristics With Cohort of Infants From all Swedish Regions Born Extremely Preterm, 2018 to 2023

**Baseline characteristics**	**Exposed group (N=474)**	**Non-exposed group (N=1510)**	**p-value**	**PS 1:1 p-value (N=425/425)**
GA, weeks (IQR)	25+4 (24+2 – 27+0)	25+6 (24+3 – 27+0)	0.05	0.17
GA 22-23 weeks, n (%)	92 (19.4%)	263 (17.4%)		
GA 24-25 weeks, n (%)	180 (38.0%)	495 (32.8%)		
GA 26-27 weeks, n (%)	202 (42.6%)	752 (49.8%)		
Birth weight, g (IQR)	755 (603-950)	780 (610-964)	0.33	0.29
Male sex, n (%)	257 (54.2%)	828 (54.8%)	0.90	0.37
Prenatal steroids given, n (%)	425 (94.0%)	1286 (90.6%)	0.03	0.26
Surfactant given, n (%)	365 (77.0%)	1129 (74.8%)	0.36	0.81
Multiple births, n (%)	99 (20.9%)	319 (21.1%)	0.92	0.21
Intubation at birth, n (%)	185 (40.0%)	626 (42.9%)	0.28	0.89

Data are presented as median (interquartile range) or number (percentage). For test between two groups Fisher's exact test was used for dichotomous variables, Chi-square test for categorical variables, and Mann-Whitney U-test for continuous variables based on the variable distribution.

Following covariates were used to create propensity score matched groups: gestational age (weeks), birth weight, Apgar score 10 min, sex, multiple births, intubation at birth, prenatal steroids, chorioamnionitis and surfactant.

GA Gestational age.

© 2026 LaMonte MJ et al. *JAMA Network Open*.

eTable 3. Statistical Evaluation of Confounders to Primary Outcome

Variable	Number of events with 'Survival without BPD at 36 weeks' PMA'	OR (95% CI)	p-value
Outborn	106 (36.8%)	1.26 (0.97 - 1.64)	0.08
Inborn (reference)	535 (31.5%)		
Male sex	317 (29.2%)	0.73 (0.61 - 0.88)	0.001
Female sex (reference)	324 (36.0%)		
Birth Weight, >median	496 (50.8%)	1.57 (1.49 - 1.65)	<.0001
<=median	303 (30.8%)		
Z-score birthweight, >median	329 (33.7%)	1.11 (1.04 - 1.18)	0.002
<=median	303 (30.8%)		
Gestational age, weeks, >median	491 (51.4%)	2.06 (1.90 - 2.24)	<.0001
<=median	150 (14.6%)		
Apgar 10 min, >median	398 (43.3%)	1.42 (1.33 - 1.51)	<.0001
<=median	211 (21.8%)		
Intubation at birth	157 (19.4%)	0.33 (0.27 - 0.40)	<.0001
No intubation at birth (reference)	469 (42.3%)		
Center affiliation, East (Reference)	181 (36.9%)		
North	52 (33.8%)	0.87 (0.60 - 1.28)	0.49
West	91 (24.5%)	0.55 (0.41 - 0.75)	0.0001
South	141 (34.0%)	0.88 (0.67 - 1.16)	0.37
South East	70 (36.3%)	0.97 (0.69 - 1.38)	0.88
Middle	106 (29.5%)	0.72 (0.54 – 0.96)	0.03
Prenatal steroids given	559 (32.7%)	1.56 (1.07 - 2.27)	0.02
No prenatal steroids given (reference)	38 (23.8%)		
Single birth (reference)	503 (32.1%)		
Multiple births, twin	125 (31.6%)	0.97 (0.77 - 1.24)	0.83
Triplet or more	13 (59.1%)	3.05 (1.29 - 7.18)	0.01
Chorioamnionitis at birth (reference)	53 (29.4%)	1.16 (0.83 - 1.62)	0.39
No chorioamnionitis at birth	588 (32.6%)		
Surfactant given at birth (reference)	399 (26.7%)	2.68 (2.17 - 3.31)	<.0001
No surfactant given at birth	242 (49.4%)		
Logistic regression was used.			

© 2026 LaMonte MJ et al. *JAMA Network Open*.

eTable 4. The Primary Outcome and its Components, With and Without Stratification and With Propensity Score–Matched Groups Analyses Presented

**Primary Outcome**	**Exposed group, N=474**	**Control group, N=632**	**OR (95% CI)**	**p-value**	**aOR (95% CI)**	**p-value**	**PS 1:1 OR (95% CI) (N=419/419)**	**p-value**
Survival without BPD	154 (32.5%)	185 (29.3%)	1.16 (0.90-1.50)	0.25	1.62 (1.16 -2.27)	0.005	1.40 (1.04-1.89)	0.03
BPD at 36 weeks PMA	233 (49.2%)	345 (54.6%)	0.80 (0.63-1.02)	0.07	0.65 (0.49-0.86)	0.002	0.73 (0.55-0.96)	0.02
Death before 36 weeks PMA	87 (18.4%)	102 (16.1%)	1.17 (0.85-1.60)	0.33	1.12 (0.75-1.69)	0.58	1.07 (0.74-1.54)	0.71
**Survival without BPD stratified by gestational age[a]**								
22-23 weeks	5 (5.4%)	4 (4.1%)	1.34 (0.35-5.14)	0.67	[b]	[b]	1.49 (0.34-6.46)	0.60
24-25 weeks	44 (24.4%)	29 (13.6%)	2.06 (1.23-3.47)	0.0062	[b]	[b]	2.51 (1.33-4.75)	0.005
26-27 weeks	105 (52.0%)	152 (47.4%)	1.20 (0.85-1.71)	0.30	1.30 (0.85-1.99)	0.23	1.37 (0.91-2.05)	0.13
**Survival without BPD stratified by birthweight deviation[a]**								
Small for gestational age	24 (24.2%)	30 (22.9%)	1.08 (0.58-1.99)	0.81	1.44 [c] (0.73-2.82)	0.29 [c]	1.37 (0.68-2.78)	0.38
Appropriate birth weight	129 (34.8%)	152 (31.0%)	1.19 (0.89-1.58)	0.24	1.62 (1.1-2.35)	0.01	1.42 (1.02-1.98)	0.04
**Survival without BPD stratified by chorioamnionitis[a]**								
Chorioamnionitis	17 (34.0%)	9 (20.0%)	2.06 (0.81-5.25)	0.13	7.78 [c] (1.72-35.14)	0.008 [c]	2.10 (0.76-5.81)	0.15
No chorioamnionitis	137 (32.3%)	176 (30.0%)	1.11 (0.85-1.46)	0.43	1.52 (1.08-2.15)	0.02	1.34 (0.98-1.84)	0.06
**Survival without BPD stratified by sex[a]**								
Female	79 (36.4%)	95 (33.2%)	1.15 (0.79-1.67)	0.46	1.57 (0.96-2.57)	0.07	1.35 (0.88-2.07)	0.17
Male	75 (29.2%)	90 (26.0%)	1.17 (0.82-1.68)	0.39	1.65 (1.03-2.64)	0.04	1.35 (0.88-2.07)	0.17

Logistic regression was used unadjusted and adjusted for covariates for overall and unadjusted for PS 1:1 matched groups.
Following covariates were used to adjust: sex, multiple births, birth weight with and without z-score, gestational age (weeks), Apgar score 10 min, intubation at birth, region of birth, prenatal steroids, and surfactant.
Following covariates were used to create propensity score matched groups: gestational age (weeks), birth weight, Apgar score 10 min, sex, multiple births, intubation at birth, prenatal steroids, chorioamnionitis and surfactant.

[a] Not adjusted for region of birth.

[b] Not enough number of events to make adjusted logistic regression analysis possible.

[c] Only adjusted for gestational age

BPD Bronchopulmonary dysplasia, *PMA* Postmenstrual age, *GA* Gestational age, *SGA* Small for gestational age, *OR* Odds ratio, *CI* Confidence Interval.

© 2026 LaMonte MJ et al. *JAMA Network Open*.

eTable 5. The Primary Outcome and Its Components, With and Without Stratification With Cohort on All Infants Born Extremely Preterm, 2018 to 2023

Primary Outcome	Exposed group, n=474	Control group, n=1510	OR (95% CI)	p-value	aOR (95% CI)	p-value	PS 1:1 OR (95% CI) (N=425/425)	p-value
Survival without BPD	154 (32.5%)	487 (32.3%)	1.01 (0.81-1.26)	0.92	1.58 (1.14 -2.20)	0.007	1.36 (1.01-1.83)	0.04
BPD at 36 weeks PMA	233 (49.2%)	748 (49.5%)	0.98 (0.80-1.21)	0.89	0.66 (0.50-0.87)	0.004	0.74 (0.56-0.97)	0.03
Death before 36 weeks PMA	87 (18.4%)	275 (18.2%)	1.01 (0.77-1.32)	0.94	1.14 (0.77-1.70)	0.52	1.09 (0.76-1.56)	0.65
**Survival without BPD stratified by gestational age[a]**								
22-23 weeks	5 (5.4%)	16 (6.1%)	0.89 (0.32-2.49)	0.82	[b]	[b]	1.99 (0.37-10.59)	0.42
24-25 weeks	44 (24.4%)	85 (17.2%)	1.56 (1.03-2.35)	0.035	[b]	[b]	2.52 (1.33-4.76)	0.004
26-27 weeks	105 (52.0%)	386 (51.3%)	1.03 (0.75-1.40)	0.86	1.30 (0.85-2.00)	0.23	1.42 (0.95-2.11)	0.09
**Survival without BPD stratified by birthweight deviation[a]**								
Small for gestational age	24 (24.2%)	73 (23.7%)	1.03 (0.61-1.75)	0.91	1.26 [c] (0.71-2.23)	0.44 [c]	1.45 (0.70-3.01)	0.32
Appropriate birth weight	129 (34.8%)	406 (34.4%)	1.02 (0.80-1.30)	0.89	1.57 (1.09-2.27)	0.015	1.39 (0.99-1.90)	0.06
**Survival without BPD stratified by chorioamnionitis[a]**								
Chorioamnionitis	17 (34.0%)	36 (27.7%)	1.35 (0.67-2.71)	0.41	1.43 [c] (0.59-3.44)	0.43 [c]	1.93 (0.68-5.44)	0.21
No chorioamnionitis	137 (32.3%)	451 (32.7%)	0.98 (0.78-1.24)	0.89	1.50 (1.07-2.12)	0.02	1.32 (0.97-1.80)	0.08
**Survival without BPD stratified by sex[a]**								
Female	79 (36.4%)	245 (35.9%)	1.02 (0.74-1.40)	0.90	1.49 (0.91-2.44)	0.12	1.45 (0.94-2.25)	0.09
Male	75 (29.2%)	242 (29.2%)	1.00 (0.73-1.36)	0.99	1.61 (1.02-2.53)	0.04	1.45 (0.94-2.25)	0.09

Logistic regression was used unadjusted and adjusted for covariates for Overall and unadjusted for PS 1:1 matched groups.
Following covariates were used to adjust: sex, multiple births, birth weight with and without z-score, gestational age (weeks), Apgar score 10 min, intubation at birth, region of birth, prenatal steroids, and surfactant.
Following covariates were used to create propensity score matched groups: gestational age (weeks), birth weight, Apgar score 10 min, sex, multiple births, intubation at birth, prenatal steroids, chorioamnionitis and surfactant.

[a] Not adjusted for region of birth.

[b] Not enough number of events to make adjusted logistic regression analysis possible.

[c] Only adjusted for gestational age

BPD Bronchopulmonary dysplasia, *PMA* Postmenstrual age, *GA* Gestational age, *SGA* Small for gestational age, *OR* Odds ratio, *CI* Confidence Interval.

eTable 6. Trend Analysis of Primary Outcome by Date of Birth

Survival without BPD and date of birth (per 1 year unit increase)	OR (CI 95%)	p-value	P-value for interaction	aOR (95% CI)	p-value	p-value for interaction
Exposed group, n=474	1.10 (0.89-1.36)	0.38	0.63	1.24 (0.94-1.63)	0.13	0.49
Control group, n=632	1.03 (0.86-1.23)	0.76		1.09 (0.87-1.37)	0.44	

Logistic regression was used unadjusted and adjusted for covariates for Overall and unadjusted for PS 1:1 matched groups.
Following covariates were used to adjust: sex, multiple births, birth weight with and without z-score, gestational age (weeks), Apgar score 10 min, intubation at birth, center of birth, prenatal steroids, and surfactant.
OR Odds ratio, *aOR* adjusted odds ratio, *CI* Confidence Interval

© 2026 LaMonte MJ et al. *JAMA Network Open*.

eTable 7. Outcome on Safety Variables With Propensity Score Matched Groups Analyses Presented

**Safety variables**	**Exposed group N=474**	**Control group N=632**	**OR (95% CI)**	**p-value**	**aOR (95% CI)**	**p-value**	**PS 1:1 OR (95%CI)**	**p-value**
Pulmonary hemorrhage	10 (2.1%)	18 (2.8%)	0.74 (0.34-1.61)	0.44	0.73 [a] (0.33-1.60)	0.43 [a]	0.61 (0.25-1.48)	0.27
Spontaneous intestinal perforation	3 (0.6%)	7 (1.1%)	0.57 (0.15-2.21)	0.42	[b]	[b]	0.50 (0.12-2.00)	0.32
Insulin treatment	111 (23.4%)	134 (21.2%)	1.14 (0.85-1.51)	0.38	1.05 (0.73-1.50)	0.79	1.14 (0.83-1.58)	0.41
Late-onset sepsis	113 (23.8%)	112 (17.7%)	1.45 (1.08-1.95)	0.01	1.30 (0.93-1.82)	0.13	1.39 (0.99-1.94)	0.06
Necrotizing enterocolitis	50 (10.5%)	53 (8.4%)	1.29 (0.86-1.93)	0.22	1.28 (0.81-2.01)	0.29	1.41 (0.88-2.25)	0.16
IVH grade 3 or 4	61 (14.0%)	83 (14.0%)	1.00 (0.70-1.43)	0.99	0.95 (0.63-1.44)	0.82	1.11 (0.73-1.68)	0.62
ROP, any grade	178 (37.6%)	234 (37.0%)	1.02 (0.80-1.31)	0.86	1.00 (0.76-1.33)	0.98	1.15 (0.87-1.52)	0.32
Treatment for ROP, grade 3-5	41 (8.6%)	54 (8.5%)	1.01 (0.66-1.55)	0.95	1.05 (0.64-1.70)	0.86	1.16 (0.72-1.88)	0.54

Logistic regression was used unadjusted and adjusted for covariates for Overall and unadjusted for PS 1:1 matched groups.
Following covariates were used to adjust: sex, multiple births, birth weight with and without z-score, gestational age (weeks), Apgar score 10 min, intubation at birth, region of birth, prenatal steroids, and surfactant.
Following covariates were used to create propensity score matched groups: gestational age (weeks), birth weight, Apgar score 10 min, sex, multiple births, intubation at birth, prenatal steroids, chorioamnionitis and surfactant.

[a] Only adjusted for gestational age due to few events.

[b] Not enough number of events to make adjusted logistic regression analysis possible.

IVH Intraventricular hemorrhage, *ROP* Retinopathy of prematurity, *OR* Odds ratio, *aOR* adjusted odds ratio, *CI* Confidence Interval, *PS* Propensity score

© 2026 LaMonte MJ et al. *JAMA Network Open*.

eTable 8. Outcome on Safety Variables With Cohort of Infants From All Swedish Regions Born Extremely Preterm, 2018 to 2023

Safety variables	Exposed group N=474	Control group N=1510	OR (95% CI)	p-value	aOR (95% CI)	p-value	PS 1:1 OR (95%CI)	p-value
Pulmonary hemorrhage	10 (2.1%)	42 (2.8%)	0.75 (0.38-1.51)	0.43	0.75 [a] (0.37-1.51)	0.43 [a]	0.80 (0.31-2.04)	0.63
Spontaneous intestinal perforation	3 (0.6%)	23 (1.5%)	0.41 (0.12-1.38)	0.15	0.41 [a] (0.12-1.36)	0.14 [a]	0.50 (0.12-2.00)	0.32
Insulin treatment	111 (23.4%)	197 (13.0%)	2.04 (1.57-2.64)	<0.001	1.06 (0.75-1.51)	0.74	1.11 (0.81-1.53)	0.52
Late-onset sepsis	113 (23.8%)	271 (17.9%)	1.43 (1.12-1.84)	0.005	1.34 (0.96-1.88)	0.08	1.31 (0.94-1.83)	0.11
Necrotizing enterocolitis	50 (10.5%)	160 (10.6%)	0.99 (0.71-1.39)	0.98	1.30 (0.83-2.04)	0.25	1.40 (0.88-2.22)	0.16
IVH grade 3 or 4	61 (14.0%)	188 (13.4%)	1.05 (0.77-1.39)	0.77	0.97 (0.64-1.45)	0.86	1.01 (0.67-1.52)	0.95
ROP, any grade	178 (37.6%)	565 (37.4%)	1.01 (0.81-1.24)	0.96	1.03 (0.78-1.36)	0.83	1.20 (0.91-1.58)	0.20
Treatment for ROP, grade 3-5	41 (8.6%)	114 (7.5%)	1.16 (0.80-1.68)	0.44	1.06 (0.65-1.72)	0.82	1.43 (0.86-2.37)	0.16

Logistic regression was used unadjusted and adjusted for covariates for Overall and unadjusted for PS 1:1 matched groups.
Following covariates were used to adjust: sex, multiple births, birth weight with and without z-score, gestational age (weeks), Apgar score 10 min, intubation at birth, region of birth, prenatal steroids, and surfactant.
Following covariates were used to create propensity score matched groups: gestational age (weeks), birth weight, Apgar score 10 min, sex, multiple births, intubation at birth, prenatal steroids, chorioamnionitis and surfactant.
[a] Only adjusted for gestational age due to few events.
IVH Intraventricular hemorrhage, *ROP* Retinopathy of prematurity, *OR* Odds ratio, *aOR* adjusted odds ratio, *CI* Confidence Interval, *PS* Propensity score

© 2026 LaMonte MJ et al. *JAMA Network Open*.
