# Supplementary material for: Early Prophylactic Hydrocortisone and Bronchopulmonary Dysplasia–Free Survival in Extremely Preterm Infants
Source: JAMA Netw Open. 2026 Feb 19;9(2):e2560146. doi: 10.1001/jamanetworkopen.2025.60146 (PMC12921520; doi:10.1001/jamanetworkopen.2025.60146)
Supplement: Supplement 2. — Data Sharing Statement [file jamanetwopen-e2560146-s002.pdf]

Data Sharing Statement

Smedbäck. Early Prophylactic Hydrocortisone, Bronchopulmonary Dysplasia, and Survival in Extremely Preterm Infants. *JAMA Netw Open*. Published February 19, 2026.
doi:10.1001/jamanetworkopen.2025.60146

Data
Data available: Yes
Data types: Deidentified participant data
How to access data: Provided upon reasonable request
When available: With publication

Supporting Documents
Document types: Statistical/analytic code
How to access documents: Provided upon reasonable request
When available: With publication

Additional Information
Who can access the data: Researchers whose proposed use of the data has been ethical approved
Types of analyses: Any research purpose with ethical approval
Mechanisms of data availability: After approval of proposal
